# Differences in the Perception Regarding Inclusion Preparation among Teachers at Different Educational Stages

**DOI:** 10.3390/ijerph20043420

**Published:** 2023-02-15

**Authors:** Natalia Triviño-Amigo, Irene Polo-Campos, Santiago Gomez-Paniagua, Sabina Barrios-Fernandez, María Mendoza-Muñoz, Jorge Rojo-Ramos

**Affiliations:** 1 Social Impact and Innovation in Health (InHEALTH) Research Group, Faculty of Sport Sciences, University of Extremadura, 10003 Cáceres, Spain; 2 BioẼrgon Research Group, University of Extremadura, 10003 Cáceres, Spain; 3 Occupation, Participation, Sustainability and Quality of Life (Ability Research Group), Nursing and Occupational Therapy College, University of Extremadura, 10003 Cáceres, Spain; 4 Research Group on Physical and Health Literacy and Health-Related Quality of Life (PHYQOL), Faculty of Sport Sciences, University of Extremadura, 10003 Cáceres, Spain; 5 Departamento de Desporto e Saúde, Escola de Saúde e Desenvolvimento Humano, Universidade de Évora, 7004-516 Évora, Portugal; 6 Physical Activity for Education, Performance and Health (PAEPH) Research Group, Faculty of Sports Sciences, University of Extremadura, 10003 Cáceres, Spain

**Keywords:** educational stage, inclusive education, perceptions, teachers, training

## Abstract

Inclusive education is fundamental, consisting of enabling all students, irrespective of their characteristics, to receive appropriate education and actively participate in school life. Teachers play an important role in this regard; thus, this study aims to analyze teachers’ perceptions regarding their preparation for inclusion by assessing possible differences depending on the educational stage (early childhood, primary, or secondary education). A total of 1098 Spanish teachers, from Extremadura, responded to three dichotomic answers about their inclusive education preparation perception and the Evaluation of Teachers’ Inclusion Readiness (CEFI-R) questionnaire, a 19-item tool composed of four dimensions: (1) conception of diversity, (2) methodology, (3) support, and (4) community participation. Pearson’s chi-square test was used to assess differences between the dichotomous questions and educational stage; Kruskal–Wallis was used to determine whether the educational stage conditioned the CEFI-R dimensions responses, and the Spearman rho was used to test the association between age groups and the CEFI-R dimensions. Statistical differences were found between secondary education and preschool education and primary education teachers in the dimensions (1) conception of diversity, (2) methodology, and (3) support. Significant differences in dimension (4) community participation between preschool education teachers and secondary and primary education teachers were found.

## 1. Introduction

The educational process should aim to increase students’ knowledge, maturity, and responsibility, as well as the democratic, equitable, and justice of communities as a whole [1,2]. This includes how functional diversity or disability has been conceived in school, and whose vision has changed over time from more paternalistic models to rights-based models. In this way, the prevailing paradigm in the past was that students with disabilities should attend special centers to receive specific attention, while in recent years the prevailing approach has been that all students should be together, and then supports should be provided in the student’s natural settings [3], as children have the right to develop friendships and to become involved in the school’s culture and daily life [4]. As a result of this exclusionary situation, some teachers, mainly those working in mainstream schools, did not tend to interact with students with disabilities [5].

Inclusive education has been placed on the global reform agenda as a result of policy advances [6,7,8,9,10]. The inclusion definition implies “inclusion as concerned with a disability” and “special educational needs (SEN)” to “an educational and social principle” [11]. In this sense, inclusive education is a commitment to include students with disabilities in mainstream schools by enhancing and adapting specific learning procedures and structures to the learner, not only by having a presence but being socially included [12,13,14]. Benefits for students with disabilities include decreased maladaptive behaviors, increased learning educational objectives, inclusive environments and social initiatives, enhanced skill acquisition and generalization, and increased friendships [15]. However, this system also has positive effects on typical students, who also benefit from being in contact with students with disabilities [16]. To promote inclusive education, several actions should be taken: teacher preparation, curriculum adaptation, collaboration with support staff, promoting positive attitudes, and students’ and families’ involvement [2,17].

However, inclusive education implementation differs widely between countries, and even between schools in the same regions [18]. Thus, educational stakeholders face challenges to promoting inclusive cultures in schools and determining how to teach inclusively [19]. Therefore, one barrier to its achievement is negative attitudes towards these students [15,20]. Other identified barriers include physical and architectural restrictions [21], inflexible curricula unable to adapt to the features of a broad spectrum of learners [22], and the lack of adequate teacher preparation [23]. Enablers would include administrative policies, supports, and leadership, and the skills of teachers and support staff in the classroom [24] since inclusive education is a multifactorial process that requires collaboration, teamwork, and effective communication between the learner’s home, school, and community to guarantee their needs are met [25].

The competencies that a teacher needs to display to meet the guidelines of inclusive education are diverse. The European Agency for Development in Special Needs Education (2011) proposes four competencies [26]: (1) valuing diversity, (2) supporting and having high expectations of students, (3) working in a team, and (4) developing the professional and personal dimension. A year later, the same agency published material outlining the competencies that an inclusive teacher should have [27]. Among other things, it highlights that during the initial training of future teachers, in addition to acquiring knowledge, they should have first-hand experience working with students with different needs and with teachers experienced in inclusive settings, since contact with people with disabilities and their reality have a great weight in inclusive preparation. To achieve inclusive and transformative education, several aspects or dimensions must be considered [28]. School leadership, a collaborative culture, adaptations of equipment and infrastructure, the possibility of sharing the experience of other teachers, and the professional development of specialists are key. Booth and Ainscow [29] highlight the need to address three dimensions: creating inclusive cultures, establishing inclusive policies, and developing inclusive practices.

Moreover, understanding how teachers perceptive their ability to implement effective techniques and methodologies [30,31] could be relevant, given that teachers’ expectations are established based on the consistency between their expectations and students’ previous performance [32,33,34,35]. In this sense, perception is the processing of external information by sensory and cognitive systems, affected mainly by sociocultural aspects and previous experiences, allowing us to generate our own beliefs about reality [36,37,38,39]. Consequently, teachers’ perception of their competence influences their choices of techniques for children with disabilities [40]. Thus, expert teachers are better than novices, integrating their knowledge of event types and learners, and are more conscious of the multidimensional complexity of teaching situations [41,42,43,44,45]. Moreover, novice teachers may have difficulty attending to and understanding some of the classroom cues [46], although research indicates that after two years of teaching in inclusive settings, these perceptions change positively [47].

Considering all of the above, the main objective of this research was to analyze Extremadura (Spain) teachers’ perception about their preparation for inclusive education, evaluating differences according to the educational stage in which they develop their teaching activity (preschool 0–6 years, primary 6–12 years, and secondary 12 and over), and to examine the possible association between this perception and their age. Therefore, it was hypothesized that the perception of teachers in this region about their preparation for inclusive education would be different according to the educational stage in which they teach (preschool 0–6 years, primary 6–12 years, and secondary 12 and over) and that there would be an association between this perception and their age. This analysis is intended to serve as an aid to establishing the starting point for public and private administrations to know the current situation to, consequently, develop future lines of action to address.

## 2. Materials and Methods

### 2.1. Participants

A nonprobability selection technique based on convenience sampling was used to collect the participants [48]. A total of 1098 active teachers from public schools in Extremadura (Spain) comprised the final sample. Table 1 shows the sociodemographic characterization of the sample, which had 16 years of teaching experience on average.

### 2.2. Procedures and Ethical Considerations

Emails and phone numbers from public schools offering preschool, primary, and/or secondary education were obtained from the Ministry of Education and Employment of the Regional Government of Extremadura (Spain) directory. After an initial contact during which the research aims were explained, an email was sent to every school to management teams to be distributed to their school’s teaching staff, which contained informed consent as well as the link to complement the tools. The instruments were distributed via Google Forms as they allow data storage in a single database, it is cost-saving, and has a higher response rate while preventing data loss [49]. It was estimated that the time to complete their participation would be about 10 min. Data were obtained between September 2021 and July 2022.

All information was gathered anonymously and kept secret. The study was performed following the Declaration of Helsinki’s principles and with the approbation from the University of Extremadura’s Bioethics and Biosafety Committee (approval code: 186/2021).

### 2.3. Instruments

Sociodemographic information was obtained using six questions: sex, age, type of contract, educational stage, school location, and years of teaching experience.

Questions about their initial and ongoing preparation: three dichotomous questions were formulated: one for initial preparation (question 1: “Do you consider that you were adequately prepared through your initial preparation to respond to the diversity of needs of your students?”) and two for ongoing preparation (question 2: “Has ongoing preparation helped you respond to the diversity of your students’ needs?” and 3: “Would you be willing to attend courses on inclusive education?”).

The Evaluation Questionnaire of Teacher Training for Inclusion, CEFI-R, was used [50] (Appendix A). This questionnaire is composed of 19 items divided into four categories: five items in dimension (1) conception of diversity, focused on ideas regarding diversity; five items in dimension (2) methodology, which assesses several facets relating to strategies to promote inclusive curricula; four items for dimension (3) supports, analyze the teacher role; and five items for dimension (4) community participation to assess educational agents’ involvement in the educational practice. A Likert-type scale with values ranging from 1 (strongly disagree) to 4 (strongly agree) was employed. The original questionnaire was in Spanish, so no translation process was necessary. Each factor > 0.70 was claimed to show good values, reporting the authors to have a 0.79 Cronbach’s alpha.

### 2.4. Statistical Analysis

Data were examined using the Statistical Package for Social Sciences (Version 26, IBM SPSS, Chicago, IL, USA). The Kolmogorov–Smirnov test was used to determine whether the data supported the assumption of normality. Nonparametric tests were chosen. In order to evaluate the internal consistency of the three dichotomous questions, the Kuder–Richardson coefficient (KR20) was calculated. Differences between the three dichotomous questions according to the educational stage were analyzed using Pearson’s chi-square test. The Kruskal–Wallis test was employed to establish the differences between the CEFI-R dimension according to the educational stage (preschool, primary, or secondary). The association between each CEFI-R dimension and age group was examined using Spearman’s rho test.

## 3. Results

The three dichotomous questions regarding teachers’ perceptions about their initial and ongoing preparation for educative inclusion are shown in Table 2. Differences were examined using the chi-square test. Differences were only found in question 2 between groups primary education and secondary education groups (*p* = 0.01) and in question 3 between preschool education and primary education groups (*p* = 0.02), preschool education and secondary education (*p* < 0.001), and primary education and secondary education groups (*p* < 0.001). Additionally, the internal consistency of the three dichotomous questions was calculated using the Kuder–Richardson coefficient (KR20) and gave an average value equal to 0.80.

Regarding the first hypothesis, which was that the perception of teachers in this region about their preparation for inclusive education would be different according to the educational stage (preschool (0–6 years), primary (6–12 years), and secondary (12 and over)), Table 3 shows CEFI-R dimension scores according to teachers’ educational level using the Kruskal–Wallis test. Significant differences were found between preschool and primary groups only in dimension 4 (*p* = 0.02); between the preschool and secondary groups significant differences were found for all dimensions (*p* = 0.03 to > 0.001); and between the primary and secondary groups only, differences were found in dimension 1 (*p* > 0.001).

Table 4 shows the correlations between the CEFI-R dimensions and age using Spearman’s rho test. An inverse relationship between questionnaire scores and age was observed for all dimensions except dimension 3. In other words, younger participants scored higher on the questionnaire in most dimensions, thus fulfilling the second hypothesis that was formulated.

## 4. Discussion

The need to understand teachers’ perceptions about their inclusive education preparation in the community of Extremadura gave rise to this study. These perceptions were examined generally for this goal, either based on their initial or ongoing training, while also examining any potential effects of the educational stage in which they develop their professional activity. Potential associations between the questionnaire dimensions and the teachers’ ages were also evaluated.

One of the greatest barriers to inclusive education according to the teachers’ opinion is the lack of initial preparation to face diversity [51], manifesting previous research that teachers have a lack of confidence or competence to instruct pupils with disabilities in inclusive settings [52]. Along these lines, one of the findings of this study showed that all groups of teachers considered that their initial training had not helped them to respond to the diversity of needs of their students (75%), with secondary education teachers being the ones who consider this lack of initial training to be the most important (78.1%). Therefore, the need to improve initial training in this respect is paramount, especially, as highlighted by several studies, in secondary teachers compared with their peers in other stages since they claim not to have sufficient pedagogical tools to program an inclusive teaching–learning process [53,54]. By contrast, Arnáiz-Sánchez reported no differences in teachers’ perception of their initial preparation for inclusion according to their educational stage [55]. This idea was supported by another study carried out in Spanish-speaking countries, where the teachers’ educational stage was not a barrier to more positive perceptions of their initial preparation for inclusive education [56]. However, these results could be conditioned by the better general inclusion perception among preschool and primary teachers compared to secondary school ones [57].

Many researchers have expressed their concern about the ongoing preparation that teachers have to teach students with disabilities [58]. In this sense, the results of this study showed better results than for initial training, as only 23.8% of the participants considered that continuous preparation had not helped them to respond to the diversity of needs of their students. These better results may be due to the willingness of primary and secondary school teachers, who consider it essential to develop effective curricula and practices to include students with disabilities [55]. However, according to Gonzalez-Gil, F. et al. [59], they argue that although research and theoretical contributions focusing on inclusive education have increased substantially, this has not led to an improvement in teacher education on inclusion, as it continues to receive marginal attention in curricula [59]. If we focus on educational stages, it is again the secondary school teachers who consider that continuous preparation has not helped them to respond to the diversity of their student’s needs (28.1%); in line with these results, other studies showed significant differences depending on the educational stage in which the teachers work and the difficulties presented by the students with whom they interact in their work [60], exposing that primary education teachers are considered to have better ongoing preparation than secondary teachers, generally due to a broader range of resources [61,62].

Thus, future teacher preparation programs, both initial and ongoing, should include collaborative abilities in addition to pedagogy and methodologies [63].

Regarding the CEFI-R dimension 1 “concept of diversity”, significant differences between the preschool and primary education teachers’ preparation were found and between primary and secondary education teachers, worsening the score as the educational stage increased. In this line, previous research showed that preschool teachers had the best perceptions towards diversity in comparison with the other educational stages [64]. Likewise, differences towards inclusion were found in preschool and primary education compared with secondary education teachers [65,66,67,68]. However, Avramidis and Norwich found more positive perceptions in secondary education teachers [69], as programs to promote inclusive education were not so often used as in the other educational stages [70].

Concerning dimensions 2 “methodology” and 3 “support”, no differences were found between primary and secondary education teachers, as well as between preschool and primary, but differences were found between secondary and preschool education teachers, similar to Orozco and Moriña [71]. Thus, the methodology effectiveness varies according to the student’s needs [72] as well as the educational stage they are enrolled [73]. Furthermore, thanks to inclusion, children without disabilities can also benefit from methodologies and supports, except in secondary education [74,75,76]. Lastly, regarding dimension 4 “community participation”, and considering that the community must be connected and affected by inclusive education since it extends beyond the educational institution walls [77], inclusive education is a tool to create inclusive societies rather than an end itself [78]. When looking for ways to make schools more inclusive, it is critical to consider all educational stakeholders’ perspectives [79]. In this sense, previous studies showed that there is more community participation in the preschool stage than in the other stages [59,63,80], due in part to the fact that students are more independent [81].

In line with our study where we found an inverse relationship between teachers’ age and perceptions towards inclusion in all four dimensions of the CEFI-R, we found that younger teachers with fewer years of experience felt more favorable towards inclusion [82,83]. This may be because younger teachers may be more familiar with the concepts of inclusion and diversity, and may be more likely to incorporate inclusive practices into their teaching. On the other hand, older teachers may have had more time to establish their teaching practices and may be more reluctant to change them. However, this is not a general rule and depends on the individual teacher’s training, experiences, and attitudes. Other studies found no differences concerning age [84], and in this regard, Chiner [85] highlighted that studies concerning perceptions of inclusion and age have led to inconclusive results.

Teacher preparation for inclusion is vitally important, as they are primarily responsible for the education of students and must be trained to meet the needs of a wide range of students, including those with disabilities and special educational needs. The results shown in this study highlight the gaps in teachers’ training and perceptions of inclusion. Therefore, this study can serve as a starting point for both individual teachers and public administrations, which should focus on actions to support teachers’ preparation for inclusion, focusing on continuous teacher education and training, especially about identifying special educational needs, planning inclusive teaching and learning, and using strategies and techniques to support students with special needs, trying to continuously evaluate and monitor progress in teachers’ preparation for inclusion and make necessary adjustments. In addition, at a general level, these results can serve as a rationale for the need to develop inclusive policies and programs that address the challenges of inclusion and provide a clear framework for inclusive education, as well as provide funding for inclusive projects that support teacher preparation and the development of inclusive classroom environments.

This study had several limitations. The sample was only from the community of Extremadura, so there may be sociodemographic variables that condition the results obtained. Likewise, it was selected using convenience sampling, so results should be interpreted with caution. It is also important to highlight the lack of previous studies evaluating these issues. Future lines should include extending the sample to a national level. Subsequent studies should consider whether teachers have had support from other institutions or specialists to meet the educational needs of pupils, as well as the preparation they have received throughout their careers.

## 5. Conclusions

The findings of this study show that concerning initial preparation, the three groups of participants mostly consider that it has not helped them to respond to the diversity of needs of their students. The secondary education group is the one that most considers that continuous preparation has not helped them to respond to the diversity of needs of their students (28.1%). Regarding whether they would be willing to attend courses on inclusive education, the higher the stage of education, the lower the interest in this topic, i.e., 96.4% would be willing to attend preschool education, compared to 88.8% in secondary education. Concerning teacher preparation for inclusion as assessed by the CEFI-R, the preschool education group was the one that obtained the best perceptions compared to the other groups, finding statistically significant differences with the secondary education group in all the dimensions; however, for the primary education group, significant differences were only found in the community participation dimension. In addition, the primary school group scored significantly higher than the secondary school group on the diversity awareness dimension. Concerning age, the results showed that the older teachers’ perceived preparedness for inclusion worsens with increasing age.

Thus, this study highlights the lack of preparation in educative inclusion to teach pupils with disabilities, regardless of the stage of education at which they work and their age. Therefore, teachers’ initial and ongoing preparation should include knowledge and strategies to ensure that all pupils have equal opportunities in the classroom and to achieve educative inclusion.

## Figures and Tables

**Table 1:** Sample characterization.

Variable	Categories	N	%
Sex	Men	350	31.9
	Women	748	68.1
Age (years)	Under 30	103	9.4
	30–40	313	28.5
	41–50	376	34.2
	Over 50	306	27.9
Type of contract	Temporary	274	25
	Permanent	824	75
Educational stage	Preschool education	221	20.1
	Primary education	471	42.9
	Secondary education	406	37
School location	Caceres	352	32.1
	Badajoz	746	67.9

**Table 2:** Dichotomous questions responses distribution according to teachers’ educational stage.

			Yes	No	*p*	*x* ^2^
Question (1) Do you think that you were properly prepared through your initial preparation to respond to the diversity of your students’ needs?						
Educational stage	Preschool education	N (%)	66 (29.9)	155 (70.1)	0.088	4.86
	Primary education	N (%)	119 (25.3)	352 (74.7)		
	Secondary education	N (%)	89 (21.9)	317 (78.1)		
	Total	N (%)	274 (25)	824 (75)		
Question (2) Has ongoing preparation helped you to respond to the diversity of your students’ needs?						
Educational stage	Preschool education	N (%)	171 (77.4)	50 (22.6)	0.031 *	6.94
	Primary education	N (%)	374 (79.4)	97 (20.6)		
	Secondary education	N (%)	292 (71.9)	114 (28.1)		
	Total	N (%)	837 (76.2)	261 (23.8)		
Question (3) Would you be willing to attend courses on inclusive education?						
Educational stage	Preschool education	N (%)	213 (96.4)	8 (3.6)	<0.001 **	39.87
	Primary education	N (%)	432 (91.7)	39 (8.3)		
	Secondary education	N (%)	330 (81.3)	76 (18.7)		
	Total	N (%)	975 (88.8)	123 (11.2)		

The correlation was significant at ** *p* < 0.01; * *p* < 0.05; *x*^2^: Pearson’s chi-square test.

**Table 3:** CEFI-R dimensions descriptive analysis and differences considering educational stage.

Dimensions	Total	Educational Stage			*p*
	Me (IQR)	Preschool Education (a)	Primary Education (b)	Secondary Education (c)	
1. Conception of diversity	3.2 (1.6)	3.2 (1)	3.2 (1.2)	3 (1.2)	(ab) (1) (ac) 0.013 (bc) >0.001
2. Methodology	3 (1.2)	3 (1)	3 (1.2)	3 (1.2)	(ab) 0.42 (ac) 0.03 (bc) 0.07
3. Supports	2.4 (1)	2.6 (0.8)	2.4 (1)	2.2 (0.8)	(ab) 0.08 (ac) >0.001 (bc) 0.12
4. Community participation	3.8 (1)	3.8 (0.8)	2.8 (1)	3.6 (1)	(ab) 0.02 (ac) 0.002 (bc) 0.92

Me = median value; IQR = interquartile range. (a) Preschool Education group; (b) Primary Education group; (c) Secondary Education group; Each score obtained is based on a Likert scale (1–4): 1 being “strongly disagree”, 2 “partially disagree”, 3 “partially agree”, and 4 “strongly agree”. (ab): *p* for differences between the preschool education and primary education groups; (ac): *p* for differences between the preschool education and secondary education groups; (bc): *p* for differences between the primary education and secondary education groups.

**Table 4:** Correlations between the dimensions and the age variable.

Dimensions	Age *ρ (p)*
(1) Conception of diversity	−0.10 (<0.001 **)
(2) Methodology	−0.03 (0.317)
(3) Supports	−0.15 (<0.001 **)
(4) Community participation	−0.16(<0.001 **)

Each score obtained on the dimensions is based on a Likert scale (1–4): 1 being “strongly disagree”, 2 “partially disagree”, 3 “partially agree”, and 4 “strongly agree”. The correlation was significant at ** *p* < 0.01.

## Data Availability

The datasets used during the current study are available from the corresponding author upon reasonable request.

## Appendix A

**Table A1:** Cuestionario para la Evaluación de la Preparación del Profesorado para la Inclusión (CEFI-R).

1 Preferiría no tener en mi aula alumnos con necesidades específicas de apoyo educativo
2 Un niño con necesidades específicas de apoyo educativo interrumpe la rutina del aula y perjudica el aprendizaje de sus compañeros
3 No debemos escolarizar alumnos con necesidades educativas especiales en centros ordinarios hasta que no tengamos la formación adecuada para ello
4 Los alumnos con necesidad específica de apoyo educativo no pueden seguir el día a día del curriculum
5 Me preocupa que mi carga de trabajo se incremente si tengo alumnos con necesidades específicas de apoyo educativo en mi clase
6 Sé cómo enseñar a cada uno de mis alumnos de manera diferente en función de sus características individuales
7 Sé cómo elaborar las unidades didácticas y las clases teniendo presente la diversidad de los estudiantes
8 Sé cómo adaptar mi forma de evaluar a las necesidades individuales de cada uno de mis alumnos
9 Sé cómo manejar y adaptar los materiales didácticos para responder a las necesidades de cada uno de mis alumnos
10 Soy capaz de adaptar mis técnicas de comunicación para asegurarme de que todos los alumnos puedan ser incluidos con éxito en el aula ordinaria
11 La planificación conjunta profesor-profesor de apoyo facilitaría que los apoyos se proporcionaran dentro del aula
12 Creo que la mejor manera de proporcionar apoyo a los alumnos es que el profesor de apoyo se incorpore al aula, en lugar de hacerlo en el aula de apoyo
13 La función del profesor de apoyo es trabajar con todo el alumnado de mi aula
14 Considero que el lugar del profesor de apoyo está dentro del aula ordinaria con cada uno de los profesores
15 El proyecto educativo debería revisarse con la participación de los distintos agentes de la comunidad educativa (profesores, padres, alumnos...)
16 Es fundamental que haya una relación muy estrecha entre el profesorado y el resto de agentes educativos (AMPA, asociación de vecinos, consejo escolar...)
17 La escuela debe fomentar la implicación de los padres y de la comunidad
18 Cada miembro del centro educativo (profesores, padres, alumnos, otros profesionales) es un elemento fundamental del mismo
19 El centro debe trabajar de forma conjunta con los recursos del barrio (biblioteca, servicios sociales, servicios sanitarios...)
